# The potential protective effect of naringenin against cadmium-induced toxicity in gingival cells

**DOI:** 10.1007/s10266-025-01187-1

**Published:** 2025-09-17

**Authors:** Mahitabe Elgamily, Bassant Mowafey, Nesreen Nabil

**Affiliations:** 1 Faculty of Dentistry, Mansoura University, Mansoura, Egypt; 2 Faculty of Dentistry, Mansoura University, Mansoura, Egypt; 3 Faculty of Dentistry, Egyptian Russian University, Cairo, Egypt

**Keywords:** Naringenin, Cadmium, MDA, SOD, Annexin V, Transmission electron microscope

## Abstract

The objective of the study was to evaluate the potential protective effect of naringenin against cadmium (Cd)-induced toxicity in human gingival cells. The safe dose of naringenin and the half-maximal inhibitory concentration (IC50) of Cd were defined using the methyl thiazolyl tetrazolium (MTT) assay. Then four groups were assigned: group I: control (cells + media), group II: cells + Cd, group III: cells + naringenin, group IV: cells  + Cd + naringenin. The cell viability was assessed by MTT assay. Lipid peroxidation (MDA), super oxide dismutase (SOD), and apoptosis by (Annexin V/PI) assay were measured. The ultrastructural changes in the gingival cells were observed by transmission electron microscopy. Cadmium decreased the viability of human gingival cells in a dose-dependent manner with an IC50 of 11.16 μM. However, naringenin at a concentration of 50 μM increased the cell viability significantly, but with higher concentrations the viability began to decrease. The exposure of the gingival cells to Cd significantly increased MDA levels and decreased the SOD. It also enhanced apoptosis in the gingival cells with subsequent ultrastructural changes such as cell blebs and nuclear fragmentation. The addition of naringenin improved the cell viability, decreased MDA, increased SOD, decreased cell apoptosis, and improved ultrastructural changes, suggesting a potential protective effect against cadmium toxicity in gingival cells. Naringenin has a potential protective effect against cadmium-induced toxicity in gingival cells.

**Graphical abstract:**

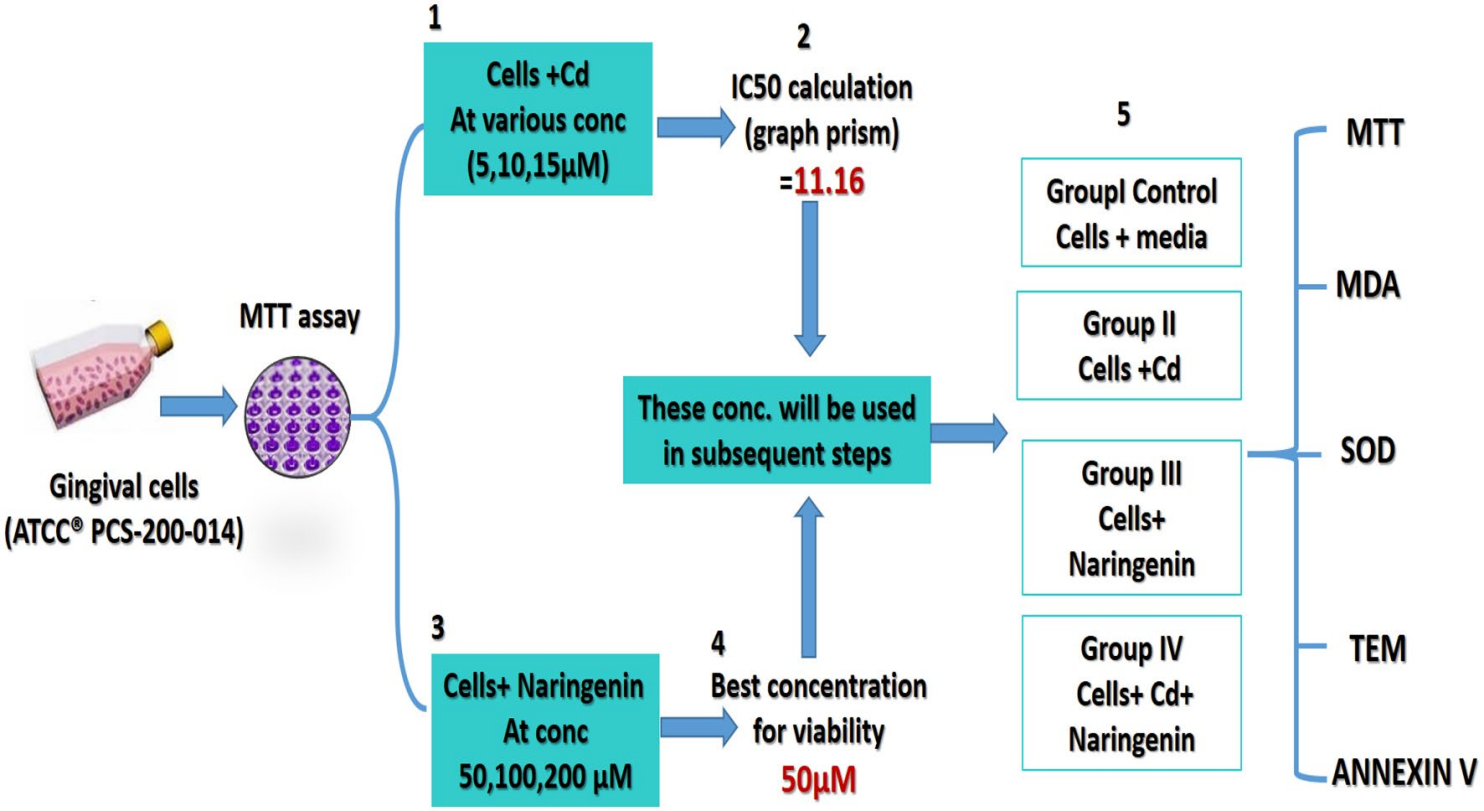

## Introduction

Cadmium (Cd) is a heavy metal, which is considered an industrial and environmental pollutant. However, it is widely used in industry such as electroplating (serving as a cathode in NK–Cd batteries) and in the fabrication of paints and dyes. The exposure of humans to Cd has increased intensely with increased environmental pollution primarily via ingestion of contaminated food and water, tobacco products, and in occupational places [1–3].

Numerous investigations have revealed that it exerts a toxic impact on human health, and as a consequence it was categorized as a member of group I carcinogens by the International Agency for Research on Cancer [4]. The liver, bone, kidney, and brain are the main parts where Cd accumulates [1, 2]. It can also worsen hearing and sight, as well as cause mutagenic and teratogenic effects [5]. Cd toxicity also reaches the salivary glands, causing apoptosis and necrosis or even autophagy in the gland cells [6]. A similar effect was noticed in the floor of the mouth of pups intoxicated with cadmium as the epithelium became thinner, with smaller and more numerous cells, indicating cellular hypotrophy [7]. Studies also revealed that it can affect the rat gingival tissue, causing dysplastic changes [8].

The oral mucosa is the primary site in which cigarette smoke and tobacco products first come into contact [9]., Cd in tobacco can accumulate in the oral mucosa through epithelial permeability [10]. Cadmium accumulation disturbs the tissues and triggers the mechanisms of cell death [6].

Cadmium can get into the cell through the ion channels and transporter systems. There are no Cd-specific ion channels or transporter proteins on the surface of the cell membrane; however, Ca2 + , Fe2 + , and Zn^2^ + ion channel systems have been found to be able to transport Cd ions through the cell membrane [11, 12].

Although the mechanisms underlying the adverse effects of Cd toxicity are still being investigated, it has been observed that it could compete with trace elements such as Fe and Zn, which are essential for metabolism [3]. Moreover, Cd indirectly induces oxidative stress by oxidizing the thiol (SH–) group of proteins, thus inhibiting their function [13, 14]. It increases reactive oxygen species (ROS) by disrupting the electron transport chain (ETC) in mitochondria, enhancing NADPH oxidase (NOX) activity, and weakening the body’s antioxidant defenses. Disrupting the (ETC) triggers a series of events including lipid peroxidation and apoptosis that further exacerbate oxidative damage and interfere with the DNA repair pathways [15]. Previous investigations have also revealed that exposure to Cd alters signal transduction pathways, resulting in an increase in cytosolic free calcium levels in multiple cell types with further obstruction of the calcium channels [16].

Flavonoid is the main group of plant polyphenols that are located in fruits, nuts, vegetables, leaves, seeds, barks of plants, and flowers. Naringenin, which belongs to the flavonoid family, is a biologically active molecule discovered in citrus fruits such as tomatoes, oranges, and grapefruits. It has several beneficial biological activities, such as antiviral activity, as it possesses an inhibitory effect against dengue virus and hepatitis C virus [17]. It also prevents the replication of chikungunya virus [18]. Naringenin exhibits antioxidant and DNA protection activities. It decreases protein carbonylation and lipid peroxidation biomarkers and enhances carbohydrate metabolism, scavenges harmful ROS, and boosts immune system activity [19].

Naringenin also shows antidiabetic, anti-inflammatory, antidepressant, antiatherogenic, hepatoprotective, nephroprotective, and antitumor activity. The antioxidant characteristics of naringenin are attributed to the presence of phenolic rings that trap electrons and scavenge peroxy radicals and superoxide anions [20]. The antioxidant activity is also attributed to the presence of the 2,3-double bond in conjugation with a 4-oxo group in the configuration of naringenin [21].

Naringenin has a wide range of effects on cells in in vitro studies in low and high doses. At 10–50 μM concentration, it acts as an antioxidant. Studies showed that NG at a concentration of 10 μM protected the fibroblast cells from toxicity by mitigating oxidative stress and metabolic pathway alterations [22]. Higher doses of 100–200 μM and even higher had an inhibitory effect on cell proliferation and an active role in the induction of apoptosis in a concentration-dependent manner, so it was studied as an anticancer drug utilizing high doses. Studies showed that naringenin is highly effective against human hepatocellular carcinoma cells and breast cancer cells as it decreased their proliferation and triggered the mitochondrial-mediated apoptosis pathway, evidenced by elevated Bax/Bcl-2 ratio, followed by the release of cytochrome C and caspase-3 activation [23, 24].

Studies documenting the adverse effects of high doses of naringenin in humans are limited. Clinical trials have tested doses up to 900 mg and concluded that doses of 150, 300, 600, and 900 mg of naringenin are safe and well tolerated in humans. At these doses, metabolites are present in circulation and are cleared within 24 h [25, 26]. Regulatory assessment toxicological studies have established a NOAEL (No Observed Adverse Effect Level) of 1320 mg/kg bw in animals when administered orally for 13 consecutive weeks or 6 consecutive months, as naringenin did not cause any real injury or toxicity to the enteric membrane. The EFSA Panel on Food Additives and Flavourings (FAF) concluded from studies carried out with naringenin that there is no concern with respect to genotoxicity and that it does not raise a safety concern, but it requested an extended one-generation toxicity study to investigate the consequence of a possible endocrine-disrupting activity [27, 28].

Many studies suggested that the use of naringenin could protect against cadmium-induced toxicity in many organs such as the liver and kidney [29, 30]. Thus, this study aims to evaluate the potential protective effect of naringenin against harmful effects caused by the exposure of the gingival cell to cadmium.

## Materials and methods

The experiments were performed at the Nile Experimental Research Center, Mansoura, Egypt, in accordance with the protocol accepted by the Mansoura University’s ethical committee; the Faculty of Dentistry, Egypt, with registration no. A19100221.

Cadmium chloride and naringenin (C15H12O5, purity > 98%) were purchased from Sigma-Aldrich, St. Louis, MO, USA.

### Maintenance of human gingival cells

Human gingival cells were obtained from ATCC® PCS-200–014. The cells were cultured according to company recommendations. The cells were preserved in DMEM F12 (DMED; Gibco, Thermo Scientific, Germany) supplemented with 20% fetal bovine serum (Life Science, UK), 100 mg/L penicillin, and 100 mg/L streptomycin (Biowest, USA) at 37 °C, 5% CO2 in an incubator (Thermo Scientific HERA cell VIOS 160i CO2 incubator with IR sensor, Waltham, USA). The experiments were performed with 70–80% confluent cells within passages 3–5.

### The cell viability assay (MTT)

The cytotoxic effect of Cd, Nar, and (Cd + Nar combination) on human gingival cells is examined by the MTT assay using Vybrant® MTT Cell Proliferation Assay Kit (V-13154). Preparation of naringenin solution was done by dissolution in dimethyl sulfoxide (DMSO) [31] while preparation of the CdCl2 working solution was done by dissolution in PBS. The gingival cells were seeded in 96-well plates and incubated for 24 h in complete culture media DMEM + 20% FBS [32]. Twenty-four hours after seeding, the complete culture media was removed and replaced by different concentrations of the tested materials for the construction of a dose–response relationship. The cells were incubated with (5, 10, 15 μM) of CdCl2 and (50,100,200 μM) of naringenin for 48 h [33].

The viability was measured after 48 h by adding 20 μL of MTT solution at a concentration of 5 mg/mL per well in phosphate-buffered saline (PBS). After overnight incubation, dissolving of the formed formazan crystals was performed by the addition of DMSO (100%), followed by scanning of the plates in a microplate reader (ELx800; BioTek Instruments, Winooski, VT) at 570 nm and Abs 690 as the reference wavelength at 37 °C in a humidified chamber to measure absorbance. Each condition was analyzed in triplicate.

The acquired data were used to calculate the IC50 for the CdCl2, utilizing software GraphPrism and to detect the  adequate  concentration of naringenin that has a positive effect on the viability of the cells. The resulting data were used in the subsequent steps.

Next, MTT test was performed for four groups after 48 h incubation: group I: control (cells in media), group II: (cells + Cdcl_2_ IC50 concentration), group III (cells + Nar adequate concentration from previous step), group IV: combination or a mix (cells + Cdcl_2_ IC50 concentration + naringenin with proper concentration) [32]. The cell viability assay was done in triplicate for each group and the viability data were obtained.

### Measurement of lipid peroxidation by malondialdehyde test (MDA)

For measurement of the MDA content, homogenization of the gingival cells of the four groups was performed, followed by centrifugation and collection of the supernatants. 200 μl supernatant was mixed with 800 μl PBS, followed by the addition of 25 μl butylated hydroxytoluene (BHT), and 500 μl trichloroacetic acid (TCA). After vortexing and incubation for 2 h on ice, the mixture was centrifuged at 2000 *g* for 15 min; then 1 ml of the supernatant was transferred into a tube containing 75 μl of 0.1 M EDTA and 250 μl of 0.05 M TBA. The tube containing the mixture was boiled in a water bath for 15 min, left to cool at room temperature, and then analyzed using a spectrophotometer at 532 nm and 600 nm wavelengths. MDA levels were calculated using a calibration curve and expressed in nmol/g tissue [34, 35].

### Superoxide dismutase (SOD) assay

The SOD assay measures enzyme activity by inhibiting phenazine methosulfate-mediated reduction of nitroblue tetrazolium dye [36]. Using an Abcam kit (Cat No. ab65354), 20 µL enzyme working solution was added to the wells containing the samples, then the wells were incubated at 37 °C for 20 min; finally, measurement of the absorbance was done at 450 nm using a Synergy H1 Hybrid BioTek microplate reader. SOD activity was then calculated as a percentage of inhibition rate [37].

### Flow cytometric analysis

The effect of Cd on gingival cell apoptosis was evaluated. The Annexin V Kit (Pharmingen FITC apoptosis, Cat. No.556547BD) is effective in the detection of both apoptotic and necrotic cells by binding FITC-labeled Annexin V to phosphatidylserine and propidium iodide (PI) to DNA. Cells from the four tested groups were washed in PBS; then apoptosis was detected using flow cytometry. In a centrifuge tube, 100 μl cell suspension from each group was washed with 1 ml PBS, followed by centrifugation at 1800 rpm for 5 min. After removal of the supernatant, the cell pellets were stained with 5 μl of annexin V–FITC and 5 μl of (PI) in 100 ml of 1 × binding buffer. The samples were incubated in the dark for 15 min, then analyzed on flow cytometry using Accuri C6 Becton Dickinson with Accuri C6 software, which analyzes the data and generates quadrant dot plots representing the viable, early, and late apoptoic cells and finally necrotic cell percentages in each group [38].

### Transmission electron microscopy

The gingival cells from the four groups were dissociated using Trypsin–EDTA followed by centrifugation, then overnight fixation with 2.5%–glutaraldehyde dissolved in 0.1 mol/L PBS at 4 °C. The specimens were then fixed with osmium tetroxide 1% in PBS at 25° C for 1 h, followed by rinsing with distilled water several times. The specimens were then dehydrated by a graded series of ethyl alcohol for 15–20 min/step, followed by 20 min.in absolute acetone. The specimens were placed at room temperature in 1:1 propylene oxide: Spurr resin for embedding. The specimens were placed in a 1.5 mL tube having Spurr resin, heated at 60 °C for more than 12 h for polymerization, and sectioned using RMC ultramicrotome. The sections were then contrasted with uranyl acetate and alkaline lead citrate and examined using a transmission electron microscope (TEM; JEOL) [39].

### Statistical methods

The collected data were analyzed by using SPSS software version 26 (SPSS, Inc., Chicago, IL, USA), presenting the results as mean and standard deviation. One-way (ANOVA) followed by post hoc Tukey test was used for comparison of quantitative parametric data across multiple different groups. *P* value less than 0.05 was considered statistically significant.

## Results

### Cell viability results (MTT assay)

To calculate the IC50 of cadmium, the effect of Cd on the viability of the gingival cells was examined at different concentrations (5, 10, 15 µM); the results clearly indicated that Cd ions lead to a cytotoxic effect in a dose-dependent manner after 48 h of exposure (0.303 ± 0.049), (0.299 ± 0.025), and (0.153 ± 0.017) respectively, with a significant difference (*P* < 0.001) (Table 1). Then, the IC50 value for Cd was calculated by (GraphPad Prism software) to be 11.16 µM.

**Table 1:** Effect of different cadmium concentrations on viability of gingival cells

Cadmium conc	5 µM	10 µM	15 µM	*P*
Viability/MTT	0.303 ± 0.049	0.299 ± 0.025	0.153 ± 0.017^ab^	< 0.001

Data expressed as mean ± SD, significance ≤ 0.05. a: significance vs conc. 5 group, b: significance vs conc. 10 group.

The effect of naringenin on cell viability was examined at concentrations 50, 100, and 200 µM; the results were (0.472 ± 0.082), (0.328 ± 0.028), and (0.136 ± 0.010) respectively. The best viability results were noticed at a concentration of 50 µM, while with 100 and 200 µM, the viability level decreased progressively with a significant difference (*P* < 0.001) (Table 2).

**Table 2:** Effect of different naringenin concentrations on the viability of gingival cells

Narengenin conc	50 µM	100 µM	200 µM	*P*
Viability/MTT	0.472 ± 0.082	0.328 ± 0.028^a^	0.136 ± 0.010^ab^	< 0.001

Data expressed as mean ± SD, significance ≤ 0.05 a: significance vs conc. 50 group, b: significance vs conc. 100 group

**Fig. 1:**
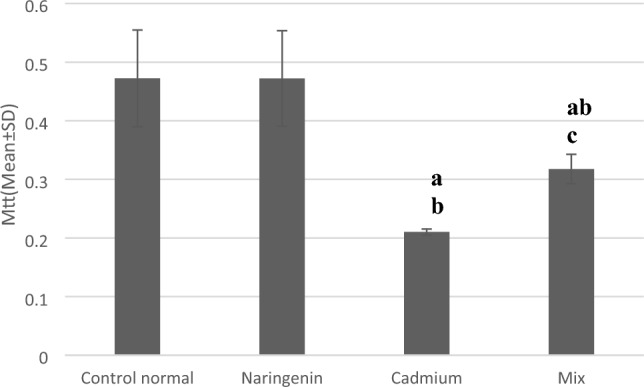
Bar chart showing effect of naringenin on cadmium-induced changes in cell viability. There was no significant difference between the cell viability of the naringenin-treated group and the control group. Cd significantly decreased the cell viability. The cell viability of the gingival cells treated with Cd and naringenin (mix) significantly enhanced in comparison with cadmium group. Data were presented as mean ± SD, significance ≤ 0.05 a: significance vs control group, b: significance vs naringenin group, c: significance vs cadmium group

When we start examining the potential protective effect of naringenin after Cd application and compare the results with the control cells or naringenin and cadmium sol application, naringenin (50 µM) showed no cytotoxic effect on the gingival cells (0.472 ± 0.082), as the results were comparable to the control group (0.472 ± 0.083) without significant difference. On the other hand, Cd (Ic 50 concentration 11.16) significantly decreased the cell viability (0.210 ± 0.005) in comparison to both control and naringenin groups (*P* < 0.001) (Table 3) (Fig. 1).

**Table 3:** Effect of naringenin on cadmium-induced changes in cell viability

	Control normal	Naringenin	Cadmium	Mix (Cd + Naringenin)	*P*
Viability/ MTT	0.472 ± 0.083	0.472 ± 0.082	0.210 ± 0.005^ab^	0.318 ± 0.025^abc^	< 0.001

Data expressed as mean ± SD, significance ≤ 0.05. a: significance vs control group, b: significance vs naringenin group, c: significance vs cadmium group

Co-treatment of Cad (IC50 concentration) and naringenin (50 µM) resulted in significant enhancement of gingival cell viability (0.318 ± 0.025) in comparison to the Cd-treated group (0.210 ± 0.005) (Table 3) (Fig. 1). The IC50 conc (11.6 µM) of Cad and naringenin at a concentration (50 µM) were used in all the following tests.

### Lipid peroxidation (MDA) results

**Fig. 2:**
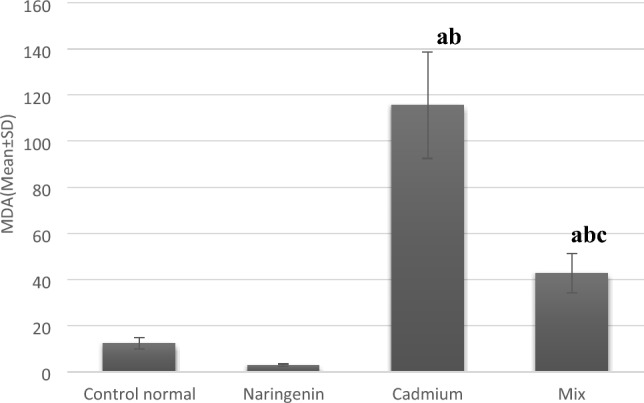
Bar chart showing effect of naringenin on cadmium-induced changes in MDA: There was no significant difference between the MDA level of the naringenin-treated group and the control group. Cadmium significantly enhanced the MDA level. Naringenin in the mix group significantly decreased the MDA level in comparison with the cadmium group. Data were presented as mean ± SD, significance ≤ 0.05. a: significance vs control group, b: significance vs naringenin group, c: significance vs cadmium group

MDA is considered a key marker of lipid peroxidation and oxidative stress. In comparison with the control group (12.37 ± 2.474), a significant rise of MDA values was noticed after exposure of the cells to Cd alone (115.6 ± 23.12) (*P* < 0.001), which indicates a marked elevation in lipid peroxidation and destruction of the cell membrane, while exposure of the cells to naringenin alone (2.88 ± 0.576) significantly decreased the lipid peroxidation (*P* < 0.001) in comparison to both control and Cd groups. Co-treatment of the gingival cells with Cd and naringenin significantly decreased the MDA (42.8 ± 8.56) in comparison with the Cd-treated group which means that treatment of the cells with naringenin significantly decreased the lipid peroxidation process, indicating a potential protective effect of naringenin against Cd-induced oxidative stress in gingival cells (Table 4) (Fig. 2).

**Table 4:** Effect of naringenin on cadmium-induced changes in the levels of malondialdehyde (MDA) and superoxide dismutase (SOD) in the gingival cells

	Control normal	Naringenin	Cadmium	Mix (Cd + naringenin)	*P*
MDA	12.37 ± 2.474	2.88 ± 0.576	115.6 ± 23.12^ab^	42.8 ± 8.56^abc^	< 0.001
SOD	331.2 ± 41.38	285.1 ± 35.63	161. ± 19.13^ab^	341.3 ± 42.66^c^	< 0.001

Data expressed as mean ± SD, significance ≤ 0.05. a: significance vs control group, b: significance vs naringenin group, c: significance vs cadmium group

### Antioxidant enzyme (SOD) results

**Fig. 3:**
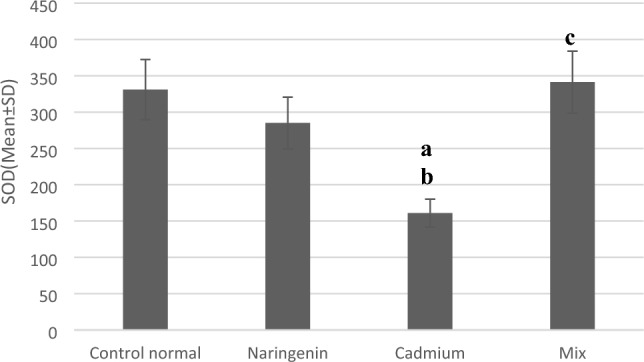
Bar chart showing effect of Naringenin on cadmium-induced changes in SOD: There was no significant difference between the SOD levels of the naringenin-treated group and the control group. Cadmium significantly decreased the SOD level. Naringenin in the mix group significantly enhanced the SOD level in comparison with cadmium group. Data were presented as mean ± SD, significance ≤ 0.05. a: significance vs control group, b: significance vs naringenin group, c: significance vs cadmium group

Regarding the antioxidant enzyme SOD, there was no significant difference between the SOD levels on naringenin-treated group (285.1 ± 35.63) and the control group (331.2 ± 41.38). On the other hand, Cd significantly decreased the SOD level (161 ± 19.13) which revealed its adverse effect on the antioxidant enzyme defense mechanisms. Combining naringenin with Cd significantly enhanced the SOD level (341.3 ± 42.66) in comparison with the Cd-treated group, which indicated that naringenin exerts excellent protection via significantly increasing the antioxidant defense enzymes (Table 4) (Fig. 3).

### Detection of apoptosis by annexin V/PI double staining results

**Fig. 4:**
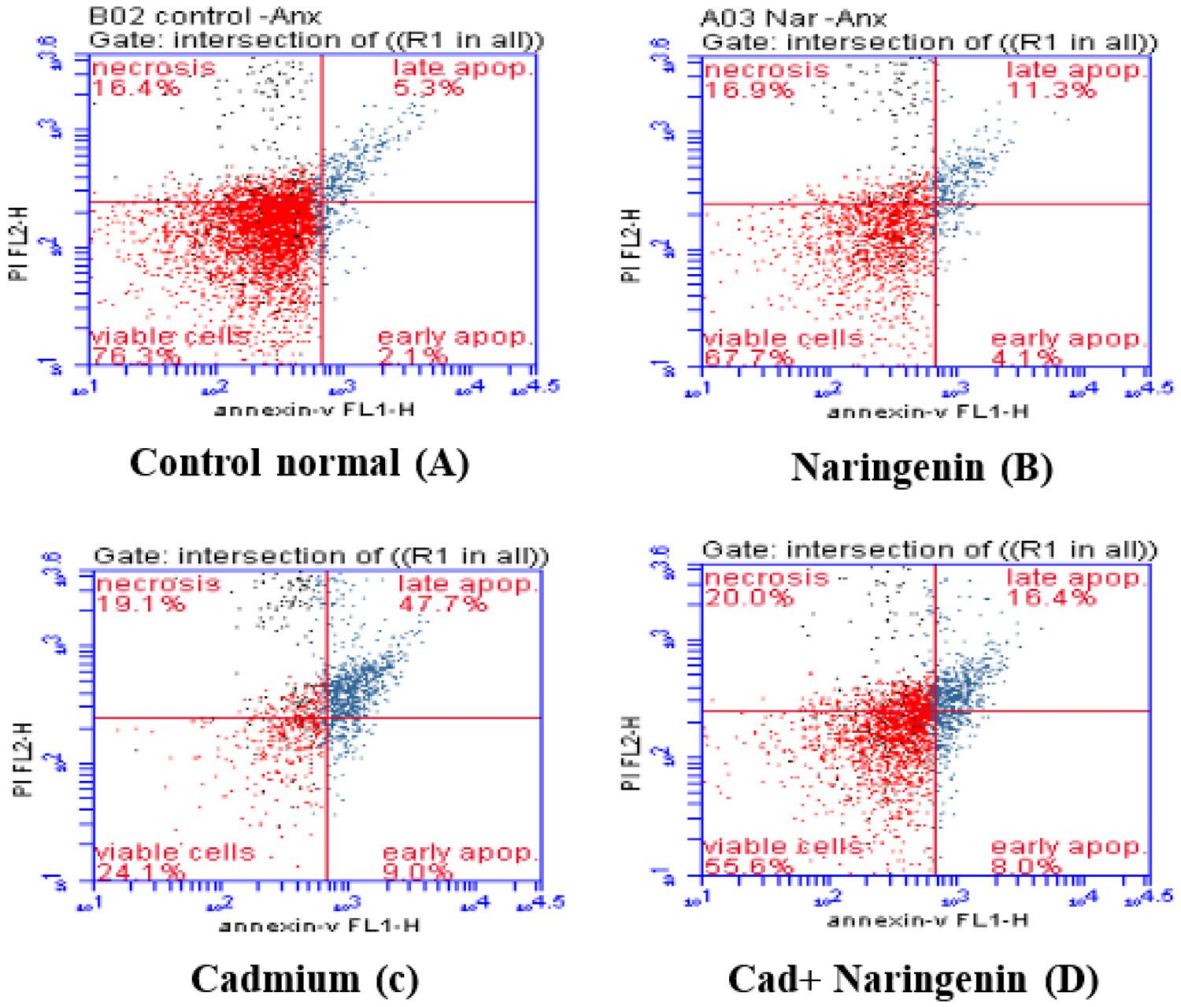
Representative flow cytometry analysis data from annexin V/PI assay. The histograms show a comparison of the distribution of annexin V/PI negative cells (**A**) and annexin V/PI positive cells after 48 h exposure to either naringenin (**B**), cadmium (**C**), both cadmium and naringenin (**D**)

**Fig. 5:**
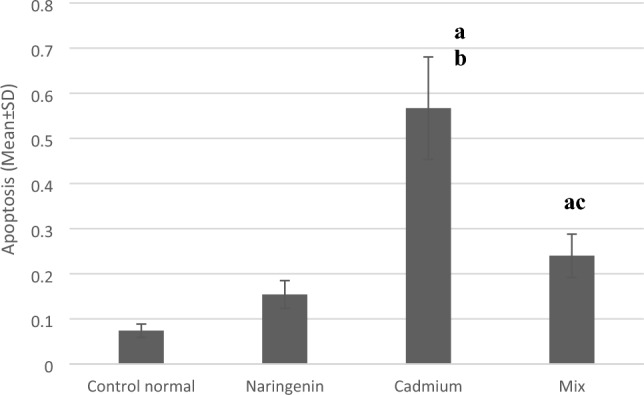
Bar chart showing the effect of naringenin on cadmium-induced apoptotic changes: There was no significant difference between the apoptosis level of the naringenin-treated group and the control group. Cadmium significantly increases the apoptosis level. Naringenin in the mix group significantly decreases the apoptosis level in comparison with cadmium group

To determine the potential protective effect of naringenin on Cd-induced apoptosis in gingival cells, flow cytometry was used to assess apoptosis in cells conjugated with annexin V–FITC/PI. Regarding the dot plot of flow cytometry, there are four quadrants: for the viable cells (left bottom), early apoptotic cells (right bottom), late apoptotic cells (right upper), and necrotic cells (upper left). The present flow cytometry figures display the results of one experiment from a series of three all yielding comparable results for each group. As shown in Figs. 4, 5, we observed that there was no significant difference between the apoptotic level of the naringenin-treated group (0.154±0.0308) and the control group (0.074±0.0148). Cadmium significantly increased the apoptosis level in the gingival cells (0.567±0.1134). By adding naringenin to Cd, it significantly decreased the apoptotic level (0.24±0.048) in comparison to the Cd-treated group (Table 5).

**Table 5:** Effect of naringenin on cadmium-induced apoptotic changes

	Control normal	Naringenin	Cadmium	Mix	*P*
Apoptosis	0.074 ± 0.0148	0.154 ± 0.0308	0.567 ± 0.1134^ab^	0.24 ± 0.048^ac^	< 0.001

Data expressed as mean ± SD, significance ≤ 0.05. a: significance vs control group, b: significance vs naringenin group, c: significance vs cadmium group

### Transmission electron microscopy results:

**Fig. 6:**
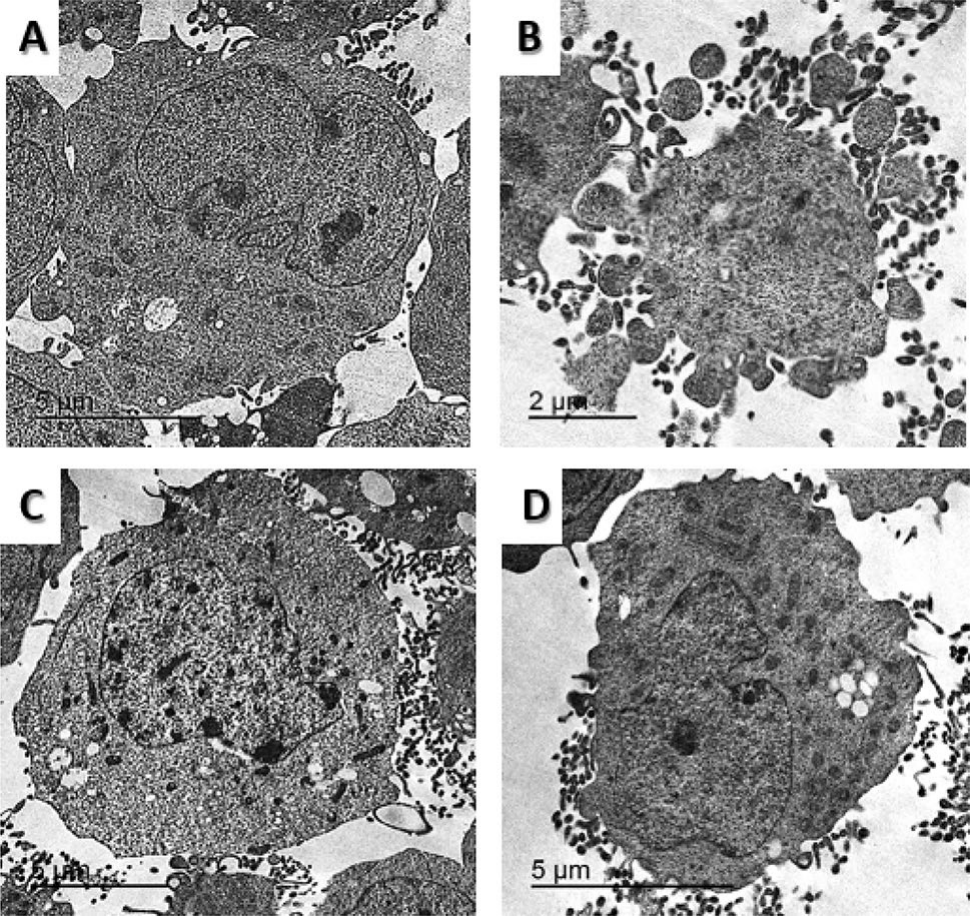
Transmission electron microscope showing effect of naringenin on cadmium-induced apoptotic changes.** A** Control gingival cells with intact nuclear and cellular membrane. **B** Cells treated with Cd with apoptotic ultrastructural changes. **C** Cells treated with naringenin showing intact nuclear and cellular membrane**. D** Cells treated with both Cd and naringenin showing intact nuclear and cellular membranes

Transmission electron microscopy showed that the control gingival cells had intact nuclear and cellular membranes, while the cells treated with Cd underwent apoptotic ultrastructural changes with typical apoptotic bodies which appeared as spherical protuberances containing fragments of chromatin clumps detached from the cell surface; the nucleus was also fragmented and disappeared. Cells treated with naringenin appeared also with intact nuclear and cellular membranes. Cells co-treated with both cadmium and naringenin appeared with intact cellular and nuclear membranes which indicates the protective effect of naringenin against the cadmium-induced destructive changes (Fig. 6).

## Discussion

Cadmium, an extremely toxic heavy metal owing to its wide environmental distribution, strong accumulation in the body, and prolonged half-life, may put the tissues and organs at risk through the generation of oxidative stress [40]. Numerous researches have testified to the use of several chelating agents to overcome Cd toxicity by boosting Cd removal, but still, chelation therapy is not specific, leading to the elimination of essential metals from the body; thus, it lacks safety and efficiency [41]. Thus, the establishment of a diet-based therapy is essential for the reduction or prevention of cadmium-induced toxicity.

In this study, the effect of naringenin on Cd-induced toxicity in human gingival cells was evaluated. The IC50 of Cd and the safe concentration of naringenin were assessed by evaluating the gingival cells’ survival rate. Cadmium induced toxicity of the gingival cells in a dose-dependent manner, which was in accordance with previous studies on human mesenchymal stem cells [42].The IC50 of Cd for gingival cells was 11.16 µM; this concentration was used for the induction of cadmium toxicity.

Also, 50, 100, and 200 µM of naringenin were used to measure the survival rate of the gingival cells by MTT. Yet, naringenin concentration above 50 µM had a negative effect on the cell survival rate, indicating that on exceeding a certain concentration, naringenin may negatively affect cells. This was in accordance with Abbas MM et al. 2019 [43], who revealed that 50 μM of naringenin resulted in greater wound closure percent than a higher concentration (100 μM), because higher doses may exert more toxic effect in normal fibroblast cell lines.

The gingival cells were then co-cultured in Cd and naringenin (50 μM) to reveal whether naringenin could improve Cd-induced cytotoxicity. The resulting MTT survival rate was significantly elevated compared to that in the Cd-treated group, which indicated that naringenin protected the gingival cells against Cd-induced toxicity. This was in accordance with Johnston JL et al. 2019, who demonstrated that preconditioning of the cells with naringenin (2.5–100 μM) exerted a cytoprotective effect that improved the Cd-mediated cell death [44]. Other studies also proved naringenin enhancement of cell viability after Cd exposure; they found that naringenin prevented the reduction of mitochondrial membrane potential and ATP levels [45]. Naringenin cytoprotection was not limited to Cd, but extends against other toxicants such as arsenate [46].

Oxidative stress is claimed to be implicated in the etiology of many diseases including infertility. Lipid peroxidation indicated by MDA level is a reflection of the destructive effect of free radicals and elevated oxidative stress. SOD is one of the major enzymes involved in the oxidase defense system and acts as a natural scavenger for reactive oxygen and superoxide anion free radicals. The enzyme can directly scavenge and eliminate free radicals, regulating the harmful attacks, preventing lipid peroxidation, and avoiding damage to the cell membrane [47, 48].

The present study revealed that Cd significantly elevates MDA content and decreases SOD activity of gingival cells; this indicates that Cd induces discrepancy and imbalance between the generation of free radicals and the antioxidant defense mechanisms, which results in the elevation of oxidative stress and destruction of cell membrane. Cadmium exerts the same effect on a variety of cell types such as piglet sterol cells and KGN cells [45, 47].

Co-supplementation of Cd and naringenin resulted in a significant elevation in SOD levels and a decline in MDA levels in the gingival cells, which means that it acts against cadmium toxicity affecting the antioxidant enzymes by preventing the accumulation of toxic metabolites and metal ions. Earlier studies reported similar findings in cadmium hepatotoxicity or with other toxicants in which naringenin reduced cisplatin-induced hepatic and renal cell toxicity and oxidative stress in rats due to the augmented activities of antioxidant enzymes. Naringenin also improved the oxidative stress exerted by Cd in KGN cells [45, 49, 50].

In the present study, flow cytometric analysis revealed that Cd induced necrosis and apoptotic cell death in gingival cells, as the cells treated with Cd demonstrated higher % of cells positive for both annexin V–FITC and PI compared to the control and naringenin‐treated group. As found in previous studies, apoptotic cell death may be because Cd generates ROS, which affects the antioxidant defense leading to DNA destruction; it also affects bioelements level as it unregulated Ca2 + homeostasis and interferes with genes and inhibits DNA repair mechanisms [48]. Another explanation is related to Cd upregulation of pro–apoptotic factors such as cytochrome c and Bax, which lead to caspase-3 mediated apoptosis [51].This apoptotic cell death was otherwise reduced significantly in the naringenin co-treatment group, indicating its protective effect. A similar effect was found on hepatic cells treated with cadmium [50].

The ultrastructural observation in the Cd-treated gingival cells showed apoptosis and necrosis with nuclear degeneration, apoptotic bodies, and vacuolization. Cadmium’s destructive effect was also in accordance with studies which reported that exposure of stomach epithelial cells to Cd resulted in ultrastructural changes such as ring-shaped or eccentrically placed nucleoli, the appearance of multiple nuclear bodies, and a segregation of the granular part of the nucleolus. They suggested that disruption of nuclear function and/or nuclear structure might be a cause of Cd toxicity [52]. Other studies indicated that cadmium-induced Sertoli piglet cells apoptosis, leading to adverse effects on spermatogenesis, which was emphasized by the appearance of morphological ultrastructural observations of chromatin condensation, apoptotic bodies, nuclear cleavage into dense bodies, and pathological vacuoles [47].

Cadmium-induced cellular destruction could be a consequence of free radical accumulation accompanied by decreased antioxidant enzyme defense and subsequent increase in lipid peroxidation and destruction of the cell membrane. Previous studies claimed cell death generated by cadmium to other several mechanisms such as endoplasmic reticulum stress that mediates cell death by affecting autophagy-related signaling, also by inducing apoptosis, pyroptosis, which is an inflammation mediated cell death, ferroptosis due to excessive iron accumulation, and autophagy [53]. In the current study, naringenin significantly reduced the cellular ultrastructure changes induced by cadmium; both cellular and nuclear membranes were preserved, no nuclear fragmentation or apoptotic bodies were observed, which indicated that naringenin has a protective effect against Cd-induced oxidative damage in gingival cells. The mechanisms contributing to naringenin’s effectiveness include the quenching of free radicals, preservation of the antioxidant defense enzymes, reduction of lipid peroxidation, its metal chelating ability, and modulation of the mitochondrial-mediated apoptotic pathway [54, 55].

## Conclusion

In conclusion, the present study demonstrated that naringenin exhibited in vitro cellular protective effects against Cd-induced gingival cell injury, as it modulates cell viability, SOD, MDA, and cellular apoptotic patterns, which lead to reduction of oxidative stress in Cd-treated cells. These outcomes indicated that the naringenin-containing dietary supplementation could help in reduction of Cd toxicity in human gingival cells. Naringenin could also be used in periodontal therapy, as it could be incorporated into topical formulations (gels or mouth rinses) for patients with cadmium exposure-related gingival inflammation. The localized delivery would achieve therapeutic concentrations while minimizing systemic exposure. Regarding occupational and environmental health, workers in cadmium-exposed industries or populations in cadmium-contaminated areas could benefit from preventive naringenin supplementation or topical oral care products as a protective measure against toxicity.

## Data Availability

The data obtained over the course of the investigation can be accessed after contacting the corresponding author.
